# Impact of *EZH2* Polymorphisms on Urothelial Cell Carcinoma Susceptibility and Clinicopathologic Features

**DOI:** 10.1371/journal.pone.0093635

**Published:** 2014-04-01

**Authors:** Yung-Luen Yu, Kuo-Jung Su, Ming-Ju Hsieh, Shian-Shiang Wang, Po-Hui Wang, Wei-Chun Weng, Shun-Fa Yang

**Affiliations:** 1 Graduate Institute of Cancer Biology, and Center for Molecular Medicine, China Medical University, Taichung, Taiwan; 2 The Ph.D. Program for Cancer Biology and Drug Discovery, China Medical University, Taichung, Taiwan; 3 Department of Biotechnology, Asia University, Taichung, Taiwan; 4 Institute of Medicine, Chung Shan Medical University, Taichung, Taiwan; 5 Cancer Research Center, Changhua Christian Hospital, Changhua, Taiwan; 6 Division of Urology, Department of Surgery, Taichung Veterans General Hospital, Taichung, Taiwan; 7 Department of Obstetrics and Gynecology, Chung Shan Medical University Hospital, Taichung, Taiwan; 8 Division of Urology, Department of Surgery, Tungs' Taichung MetroHarbor Hospital, Taichung, Taiwan; 9 Department of Medical Research, Chung Shan Medical University Hospital, Taichung, Taiwan; Sudbury Regional Hospital, Canada

## Abstract

**Background:**

The gene *EZH2*, the polycomb group protein enhancer of zeste 2, encodes a transcriptional repressor that also serves as a histone methyltransferase that is associated with progression to more advanced disease in a variety of malignancies. *EZH2* expression level in urothelial cell carcinoma (UCC) is highly correlated with tumor aggressiveness, but it has not been determined if specific *EZH2* genetic variants are associated with UCC risk. This study investigated the potential associations of *EZH2* single-nucleotide polymorphisms with UCC susceptibility and its clinicopathologic characteristics.

**Methodology/Principal Findings:**

A total of 233 UCC patients and 552 cancer-free controls, all of whom were from Taiwan, were analyzed for four *EZH2* single-nucleotide polymorphisms (rs6950683, rs2302427, rs3757441, and rs41277434) using real-time PCR genotyping. After adjusting for other co-variants, we found that individuals carrying at least one C allele at *EZH2* rs6950683 had a lower risk of developing UCC than did major allele carriers. The CCCA or TGTA haplotype among the four *EZH2* sites was also associated with a reduced risk of UCC. Furthermore, UCC patients who carried at least one G allele at rs2302427 had a lower invasive tumor stage than did patients carrying the major allele.

**Conclusions:**

The rs6950683 SNPs of *EZH2* might contribute to the prediction of UCC susceptibility. This is the first study to provide insight into risk factors associated with *EZH2* variants in carcinogenesis of UCC in Taiwan.

## Introduction

The urothelium is the epithelial lining of the urinary tract from the renal calyces to the bladder. More than 95% of all bladder cancers are urothelial cell carcinomas (UCC). The majority of UCCs are bladder tumors, whereas upper urinary tract tumors and tumors of the urethra contribute less than 10%. Bladder cancer is an excellent model for studying genetic susceptibility and gene–environment interaction (e.g., gene–smoking and gene–occupational exposure interactions) in cancer etiology [1]. Furthermore, epidemiological studies indicate that UCC has a familial component with an almost 2-fold increased risk among first degree relatives of patients with UCC, which cannot be explained by smoking, suggest that genetic (heritable) factors are important in development of UCC of the bladder [2].

UCC is a highly aggressive malignancy that causes substantial morbidity and mortality [3]. The major clinical distinction is between the non-muscle invasive Ta and T1 tumors and the muscle invasive T2–T4 tumors [4]. The molecular biology of UCC has been extensively studied in recent years, and many genetic alterations and modified expression patterns of certain oncogenes and tumor suppressor genes have been linked to its tumorigenesis and progression [5].

Epigenetic changes by DNA methylation at CG dinucleotide sites (CpGs) are frequent events in tumor development [6], [7]. Most differential DNA methylation has been attributed to genes that are essential for developmental processes, often polycomb repressive complex 2 (PRC2)–regulated genes [4], [8], [9]. The enhancer of zeste homolog 2 (EZH2) gene, known as a member of the polycomb group of genes, has been found to contribute to the maintenance of cell identity, cell cycle regulation and oncogenesis [10]. The EZH2 is a SET domain containing methyltransferase catalyzing the methylation of histone H3, forming the transcriptional repressive epigenetic mark H3K27me3 [9]. Recently, EZH2 was linked to the aggressiveness of human cancers, including breast cancer [11] and prostate cancer [12]. Overexpression of EZH2 correlates with advanced stages of human cancer progression and poor prognosis [13]. In addition, EZH2 promotes the epithelial–mesenchymal transition, a process that is associated with cancer progression and metastasis [14].

Single-nucleotide polymorphisms (SNPs) are the most common type of DNA sequence variation which influences the progression of gene-related diseases [15]. Epidemiological studies suggest that SNPs are important in mediating an individual's susceptibility to many types of cancer [10], [16]. Although gene polymorphisms of EZH2 are associated with several types of cancer [16]–[18], the association between *EZH2* variants and UCC risk and prognosis has been poorly investigated. We therefore performed a case-control study of four SNPs located in the promoter, exonic, and intronic regions of *EZH2* to assess the associations between these SNPs and UCC susceptibility and clinicopathologic characteristics.

## Materials and Methods

### Study subjects and specimen collection

This hospital-based case-control study recruited 233 UCC patients between 2010 and 2012 at the Taichung Veterans General Hospital in Taichung, Taiwan. The diagnosis of UCC was made according to the criteria specified in the national guidelines for UCC. During the same study period, 552 ethnic group-matched individuals were enrolled as the controls that entered the physical examination. These control groups had neither self-reported history of cancer of any sites. Personal information and characteristics collected from the study subjects using interviewer-administered questionnaires contained questions involving demographic characteristics and the status of cigarette smoking.

UCC patients were clinically staged at the time of diagnosis according to the tumor/node/metastasis staging system of the American Joint Committee on Cancer (2002) [16]. The patients' clinicopathological characteristics, including clinical staging, lymph node metastasis, and histopathologic grading levels, were verified by chart review. Whole-blood specimens collected from the controls and UCC patients were placed in tubes containing EDTA, immediately centrifuged, and stored at −80°C. Before commencing the study, approval was obtained from the Institutional Review Board of Taichung Veterans General Hospital, and informed written consent was obtained from each individual.

### Selection of *EZH2* Polymorphisms

A total of four SNPs in *EZH2* (NM_004456) were selected from the International HapMap Project data for this study. We included the non-synonymous SNP rs2302427 (D185H in exon 6) in the coding sequence of the gene. The other SNPs (rs6950683, rs3757441, and rs41277434) were selected in this study because the gene polymorphisms of these SNPs have been found to associate with colon and lung cancers [17], [18].

### Genomic DNA extraction

Genomic DNA was extracted using QIAamp DNA blood mini kit reagents (Qiagen, Valencia, CA). DNA was dissolved in buffer containing 10 mM Tris (pH 7.8) and 1 mM EDTA and then quantified by measurement of the optical density at 260 nm. Final DNA preparations were stored at −20°C and used as templates for PCR [19], [20].

### Real-time PCR

Allelic discrimination of the *EZH2* polymorphisms rs6950683, rs2302427, rs3757441, and rs41277434 was assessed using an ABI StepOneTM Real-Time PCR System (Applied Biosystems), SDS v3.0 software (Applied Biosystems), and the TaqMan assay. The final volume for each reaction was 5 μL, containing 2.5 μL TaqMan Genotyping Master Mix, 0.125 μL TaqMan probes mix, and 10 ng genomic DNA. The reaction conditions included an initial denaturation step at 95°C for 10 min followed by 40 cycles at 95°C for 15 sec and 60°C for 1 min [21], [22]. For each assay, appropriate controls (nontemplate and known genotype) were included in each typing run to monitor reagent contamination and as a quality control. To validate results from real-time PCR, around 5% of assays were repeated and several cases of each genotype were confirmed by the DNA sequence analysis.

### Statistical analysis

Hardy–Weinberg equilibrium was assessed using a χ^2^ goodness-of-fit test for biallelic markers. A Mann-Whitney U-test and a Fisher's exact test were used to compare differences of age and demographic characteristics distributions between controls and UCC patients. The odds ratios (ORs) with 95% confidence intervals (CIs) were estimated by logistic regression models. The adjusted odds ratios (AORs) with 95% CIs of the association between genotype frequencies and UCC risk as well as clinical pathological characteristics were estimated by multiple logistic regression models after controlling for other covariates. The haplotype-based analysis was carried out using the PHASE version 2.1. All *p* values were calculated use the standard Bonferroni threshold for the four SNPs and a value of <0.0125 was considered statistically significant. The data were analyzed using SAS statistical software (Version 9.1, 2005; SAS Institute Inc., Cary, NC).

## Results

This study analyzes the demographic characteristics of sample specimens and found that there were significantly different distributions of age (control: 51.65±14.62; UCC: 68.55±11.82; *p*<0.001) and gender (*p*<0.001) between controls and UCC patients (Table 1). To reduce possible interference of confounding variables, AORs with 95% CIs were estimated by multiple logistic regression models after controlling for age and gender in each comparison. Table 2 shows the genotype distributions and the association between UCC and *EZH2* polymorphisms. In our recruited control group, the frequencies of *EZH2* polymorphisms (rs3757441 and rs41277434) were in Hardy-Weinberg equilibrium, except for rs6950683 (χ^2^ value: 5.59) and rs2302427 (χ^2^ value: 5.77). Furthermore, after reconstructing linkage disequilibrium (LD) plot of the four SNPs, we determined one observed haploblock in which rs6950683 and rs2302427 showed high linkage disequilibrium in our study (D' = 0.96). The alleles with the highest distribution frequency at *EZH2* rs6950683, rs2302427, rs3757441, and rs41277434 in both UCC patients and controls were homozygous T/T, homozygous C/C, homozygous T/T, and homozygous A/A, respectively. Individuals carrying TC+CC at rs6950683 showed a 0.565-fold (95% CI: 0.382–0.835) lower risk of UCC, and those carrying CG, GG, or CG+GG at rs2302427 showed a 0.683-fold (95% CI: 0.480–0.972), 0.388-fold (95% CI: 0.168–0.895) and a 0.624-fold (95% CI: 0.412–0.944) lower risk of UCC, respectively, compared with individuals carrying the major allele. Individuals with polymorphisms at rs3757441 and rs41277434 showed no reduction in UCC risk compared with major allele individuals.

**Table 1:** Demographic characteristics of controls and patients with UCC.

Variable	Controls (*N* = 552)	Patients (*N* = 233)	*p* value
**Age (years)**	**Mean ± S.D.**	**Mean ± S.D.**	
	51.65±14.62	68.55±11.82	<0.001
**Gender**	***n*** ** (%)**	***n*** ** (%)**	
Male	449 (81.3%)	149 (63.9%)	
Female	103 (18.7%)	84 (36.1%)	<0.001
**Tobacco consumption**			
No	336 (60.9%)	166 (71.5%)	
Yes	216 (39.1%)	67 (28.8%)	0.006
**Stage**			
Superficial tumor (pTa–pT1)		142 (60.9%)	
Invasive tumor (pT2–pT4)		91 (39.1%)	
**Tumor T status**			
T0		65 (27.9%)	
T1–T4		168 (72.1%)	
**Lymph node status**			
N0		212 (91.0%)	
N1+N2		21 (9.0%)	
**Metastasis**			
M0		229 (98.3%)	
M1		4 (1.7%)	
**Histopathologic grading**			
Low grade		36 (15.5%)	
High grade		197 (84.5%)	

Mann-Whitney U test or Fisher's exact test was used to determine the significance of differences between healthy controls and patients with UCC.

**Table 2:** Distribution frequency of *EZH2* genotypes in controls and patients with UCC.

Variable	Controls (*N* = 552) *n* (%)	Patients (*N* = 233) *n* (%)	OR (95% CI)	AOR (95% CI)^a^
**rs6950683**				
TT	264 (47.8%)	130 (55.8%)	1.00	1.00
TC	220 (39.9%)	83 (35.6%)	0.766 (0.552–1.064) *p* = 0.112	0.749 (0.538–1.043) *p* = 0.108
CC	68 (12.3%)	20 (8.6%)	0.597 (0.348–1.026) *p* = 0.062	0.597 (0.347–1.028) *p* = 0.086
TC+CC	288 (52.2%)	103 (44.2%)	0.726 (0.534–0.988) *p* = 0.041	0.565 (0.382–0.835) *p* = 0.004^b^
**rs2302427**				
CC	346 (62.7%)	169 (72.5%)	1.00	1.00
CG	171 (31.0%)	57 (24.5%)	0.682 (0.480–0.970) *p* = 0.033	0.683 (0.480–0.972) *p* = 0.034
GG	35 (6.3%)	7 (3.0%)	0.409 (0.178–0.941) *p* = 0.035	0.388 (0.168–0.895) *p* = 0.038
CG+GG	206 (37.3%)	64 (27.5%)	0.636 (0.455–0.890) *p* = 0.008	0.624 (0.412–0.944) *p* = 0.026
**rs3757441**				
TT	271 (49.1%)	123 (52.8%)	1.00	1.00
TC	223 (40.4%)	88 (37.8%)	0.869 (0.628–1.205) *p* = 0.400	0.754 (0.500–1.137) *p* = 0.178
CC	58 (10.5%)	22 (9.4%)	0.836 (0.489–1.427) *p* = 0.511	0.650 (0.334–1.265) *p* = 0.205
TC+CC	281 (50.9%)	110 (47.2%)	0.862 (0.635–1.172) *p* = 0.344	0.730 (0.497–1.072) *p* = 0.108
**rs41277434**				
AA	517 (93.6%)	218 (93.6%)	1.00	1.00
AC	34 (6.2%)	15 (6.4%)	1.046 (0.558–1.960) *p* = 0.888	0.683 (0.319–1.461) *p* = 0.325
CC	1 (0.2%)	0 (0%)	----	----
AC+CC	35 (6.4%)	15 (6.4%)	1.016 (0.544–1.899) *p* = 0.959	0.651 (0.305–1.388) *p* = 0.267

The odds ratios (ORs) and their 95% confidence intervals (CIs) were estimated by logistic regression models.

a The adjusted odds ratios (AORs) with their 95% CIs were estimated by multiple logistic regression models after controlling for age and gender.

b Use the standard Bonferroni threshold for the four SNPs (*p*<0.0125).

The distribution of clinical status/*EZH2* genotypes in UCC patients was estimated to clarify the role of *EZH2* polymorphisms in the clinicopathologic state of UCC patients. Clinical status assessments included tumor/node/metastasis staging, lymph node status, metastasis, and histopathologic grading. Compared with control subjects having the major allele, patients with at least one C allele at *EZH2* rs6950683 showed no significant differences between *EZH2* genotypic frequencies and clinicopathological variables (Table 3); however, patients with at least one G allele at *EZH2* rs2302427 showed a 0.418-fold (95% CI: 0.220–0.794) decrease in invasive tumor stage (Table 4). No significant differences were observed between other *EZH2* genotypic frequencies and any clinicopathological variables (data not shown).

**Table 3:** Distribution frequency of the clinical status and of the *EZH2* rs6950683 genotype in patients with UCC.

	Genotypic frequency			
Variable	TT (*N* = 130) *n* (%)	TC+CC (*N* = 103) *n* (%)	OR (95% CI)	*p* value
**Stage**				
Superficial tumor (pTa–pT1)	82 (63.1%)	60 (58.3%)	1.00	
Invasive tumor (pT2–pT4)	48 (36.9%)	43 (41.7%)	1.224 (0.721–2.079)	0.453
**Tumor T status**				
T0	37 (28.5%)	28 (27.2%)	1.00	
T1–T4	93 (71.5%)	75 (72.8%)	1.066 (0.598–1.899)	0.829
**Lymph node status**				
N0	120 (92.3%)	91 (89.3%)	1.00	
N1+N2	10 (7.7%)	11 (10.7%)	1.435 (0.584–3.523)	0.429
**Metastasis**				
M0	129 (99.2%)	100 (97.1%)	1.00	
M1	1 (0.8%)	3 (2.9%)	3.870 (0.397–37.767)	0.211
**Histopathologic grading**				
Low grade	21 (16.2%)	15 (14.6%)	1.00	
High grade	109 (83.8%)	88 (85.4%)	1.130 (0.550–2.321)	0.739

**Table 4:** Distribution frequency of the clinical status and of the *EZH2* rs2302427 genotype in patients with UCC.

	Genotypic frequency			
Variable	CC (*N* = 169) *n* (%)	CG+GG (*N* = 64) *n* (%)	OR (95% CI)	*p* value
**Stage**				
Superficial tumor (pTa–pT1)	94 (55.6%)	48 (75.0%)	1.00	
Invasive tumor (pT2–pT4)	75 (44.4%)	16 (25.0%)	0.418 (0.220–0.794)	0.007
**Tumor T status**				
T0	42 (24.9%)	23 (35.9%)	1.00	
T1–T4	127 (75.1%)	41 (64.1%)	0.590 (0.318–1.094)	0.092
**Lymph node status**				
N0	150 (88.8%)	62 (96.9%)	1.00	
N1+N2	19 (11.2%)	2 (3.1%)	0.255 (0.058–1.126)	0.053
**Metastasis**				
M0	165 (97.6%)	64 (100%)	1.00	
M1	4 (2.4%)	0 (0%)	---	0.214
**Histopathologic grading**				
Low grade	25 (14.8%)	11 (17.2%)	1.00	
High grade	144 (85.2%)	53 (82.8%)	0.836 (0.385–1.817)	0.652

The haplotype distributions of *EZH2* rs6950683, rs2302427, rs3757441, and rs41277434 were further evaluated, and eight haplotypes were derived from these four SNPs in our recruited individuals. The most common haplotype in the control group was TCTA (42.4%), and it was, therefore, chosen as the reference. Compared with this reference, two minor haplotypes, CCCA and TGTA, significantly reduced the risk of UCC by 0.674-fold (95% CI: 0.521–0.871) and 0.489-fold (95% CI: 0.357–0.671), respectively (Table 5).

**Table 5:** Distribution frequency of *EZH2* haplotype in controls and UCC patients.

Variable				Controls (*N* = 1104) *n* (%)	Patients (*N* = 466) *n* (%)	OR (95% CI)	*p* value
rs6950683T/C	rs2302427C/G	rs3757441T/C	rs41277434 A/C				
T	C	T	A	468 (42.4%)	255 (54.7%)	Reference	
C	C	C	A	335 (30.3%)	123 (26.4%)	0.674 (0.521–0.871)	0.003
T	G	T	A	240 (21.7%)	64 (13.7%)	0.489 (0.357–0.671)	<0.001
T	C	T	C	36 (3.3%)	15 (3.3%)	0.765 (0.411–1.423)	0.396
Others#				25 (2.3%)	9 (1.9%)	0.661 (0.304–1.437)	0.293

# Others: CCTA (20), TGCA (7), TCCA (6), CGTA (1).

## Discussion

Epigenetic gene regulation has evolved as a key mechanism contributing to cell-cycle control and cell-fate determination [23], [24]. The polycomb group proteins function in an epigenetic regulatory system associated with gene silencing [5]. EZH2 plays an important role in cell-cycle regulation, and its gene has emerged as a novel oncogene and putative target for therapy [10]. Previous studies have suggested that EZH2 expression is high in UCC tumors with advanced stage and higher grades [25], [26]. Therefore, *EZH2* polymorphisms may be associated with UCC development. However, the predictive value of *EZH2* for susceptibility to UCC has not previously been investigated.

SNPs in genes encoding cancer susceptibility factors have been documented to influence gene expression, protein function, and disease susceptibility in certain individuals [27], [28]. We performed a genetic association analysis of *EZH2* variants with UCC. *EZH2* contains 20 exons, 19 introns, and 41 identified SNPs [29] and encodes two isoforms of different transcript sizes [18]. In our present hospital-based case-control study, four *EZH2* SNPs were genotyped in 233 patients with UCC and 552 healthy controls. We observed that SNPs rs6950683 and rs2302427 were associated with reduced UCC risk (Table 2).

SNP rs6950683 is located in a tissue-specific CpG island within the *EZH2* promoter region upstream of the *EZH2* coding sequence. Thus, it may impact gene expression by affecting promoter function. SNP rs2302427 is located in exon 6 and causes a non-synonymous amino-acid change from aspartic acid to histidine, which might affect the protein function. Further functional studies are needed to confirm the specific mechanisms by which these *EZH2* polymorphisms influence UCC development.

In recent years, haplotype-based association studies have identified genetic variants that predict cancer predisposition [10], [30]. Although many SNPs have no direct effect on gene products, they can still be used as genetic markers to locate adjacent functional variants that contribute to disease. When each SNP involved the haplotypes has a true contribution to disease susceptibility, haplotype analysis provides greater statistical power. Therefore, haplotype analysis is sometimes advantageous over analysis of individual SNPs for detecting an association between alleles and a disease phenotype [23]. Our haplotype analysis of the four *EZH2* SNPs rs6950683, rs2302427, rs3757441, and rs41277434 revealed that the CCCA and TGTA haplotypes were associated with a lower risk of UCC (Table 5). However, it is possible that these *EZH2* SNPs are linked with other functional polymorphisms and are, therefore, not directly responsible for the decreased susceptibility to UCC.S

One of the weak points of our study is missing HWE. This study revealed the nonconformity of *EZH2* rs6950683 (χ^2^ value: 5.59) and rs2302427 (χ^2^ value: 5.77) genotypes to Hardy-Weinberg equilibrium in the control group. A previous study with 107000 genotypes generated from 443 SNPs has found that genotype distributions for 36 out of 313 assays (11.5%) were deviated from HWE (*p*<0.05) [31]. Although the reason for the nonconformity of *EZH2* genotypes to HWE in our control group is not explored yet, results from the abovementioned study may provide some direction for our future study.

To the best of our knowledge, this is the first study to associate *EZH2* polymorphisms with risk of UCC. Results showed two SNPs of *EZH2* decreased UCC susceptibility, and UCC patients with the polymorphism rs2302427 had a lower risk of invasive tumor stage. These results suggest that *EZH2* variants might be novel susceptibility markers linked to UCC. However, the number of case and control subjects in this study was relatively small, and thus additional studies with larger sample sizes are needed to validate the relevance of *EZH2* polymorphisms to UCC. Further investigations should focus on *EZH2* sequence variants and their biological function in UCC patients.
